# Spontaneous intracranial hypotension presenting as thunderclap headache: a case report

**DOI:** 10.1186/s13104-015-1068-1

**Published:** 2015-03-29

**Authors:** Thashi Chang, Chaturaka Rodrigo, Lasitha Samarakoon

**Affiliations:** Department of Clinical Medicine, Faculty of Medicine, University of Colombo, 25, Kynsey Road, Colombo 08, Sri Lanka; University Medical Unit, National Hospital of Sri Lanka, Colombo, Sri Lanka

**Keywords:** Thunderclap headache, Spontaneous intracranial hypotension, Meningeal enhancement

## Abstract

**Background:**

Spontaneous intracranial hypotension is a rare but treatable cause of a disabling headache syndrome. It is characterized by positional orthostatic headache, pachymeningeal enhancement and low cerebrospinal fluid pressure. However, the spectrum of clinical and radiographic manifestations is varied and misdiagnosis is common even in the modern era of magnetic resonance imaging. Spontaneous intracranial hypotension presenting as thunderclap headache is recognized but rare.

**Case presentation:**

A 41-year-old Sri Lankan female presented with thunderclap headache associated with nausea and vomiting, but the headache was characterized by positional variation with aggravation in the upright posture and relief in the supine posture. Gadolinium-enhanced cranial magnetic resonance imaging demonstrated generalized meningeal enhancement and normal magnetic resonance angiography while lumbar puncture revealed a cerebrospinal fluid opening pressure of less than 30 millimetres of water. Magnetic resonance myelography failed to identify the site of cerebrospinal fluid leak. The patient was managed conservatively with bed-rest, intravenous hydration, analgesics and an increased intake of oral coffee which led to a gradual relief of headaches in the upright posture.

**Conclusions:**

Spontaneous intracranial hypotension can rarely present as thunderclap headache. Awareness of its varied spectrum of presentations would avoid inappropriate investigations, misinterpretation of imaging results and ineffective treatment.

## Background

Spontaneous intracranial hypotension (SIH) (first described by Schaltenbrand) [1] is an important cause of headache that is often underdiagnosed. It has an estimated annual incidence of 5 per 100 000, peaking around the age of 40 and affects women more frequently than men [2]. Orthostatic headache, low cerebrospinal fluid (CSF) pressure, and diffuse meningeal enhancement on brain magnetic resonance imaging (MRI) are the major features of the classic syndrome. However, the spectrum of clinical and radiographic manifestations is varied, with diagnosis largely dependent on clinical suspicion [3]. We describe a patient presenting with typical clinical and radiological features of SIH and review the salient literature.

## Case presentation

A 41-year-old, previously healthy Sri Lankan female presented with sudden onset severe headache for one day. The headache started in the occipital region and spread towards the vertex. It worsened with standing and was accompanied with nausea and vomiting. The patient described it as her ‘worst-ever’ headache. She denied a past history of migraine. Remarkably, the headache resolved with lying supine and recurred on sitting up or standing. It would commence as a sensation of ‘heaviness’ of her head that would gradually progress to a severe, disabling headache. The maximum duration that she could tolerate an upright posture was approximately one hour. She did not have any other co-morbidities and denied use of any medicinal or recreational drugs. There was no history of surgery or trauma involving the head, neck or spine.

On examination, she was comfortable in the supine position and detested sitting up or standing. The cardiovascular, respiratory, abdominal and nervous system examinations were normal.

**Figure 1:**
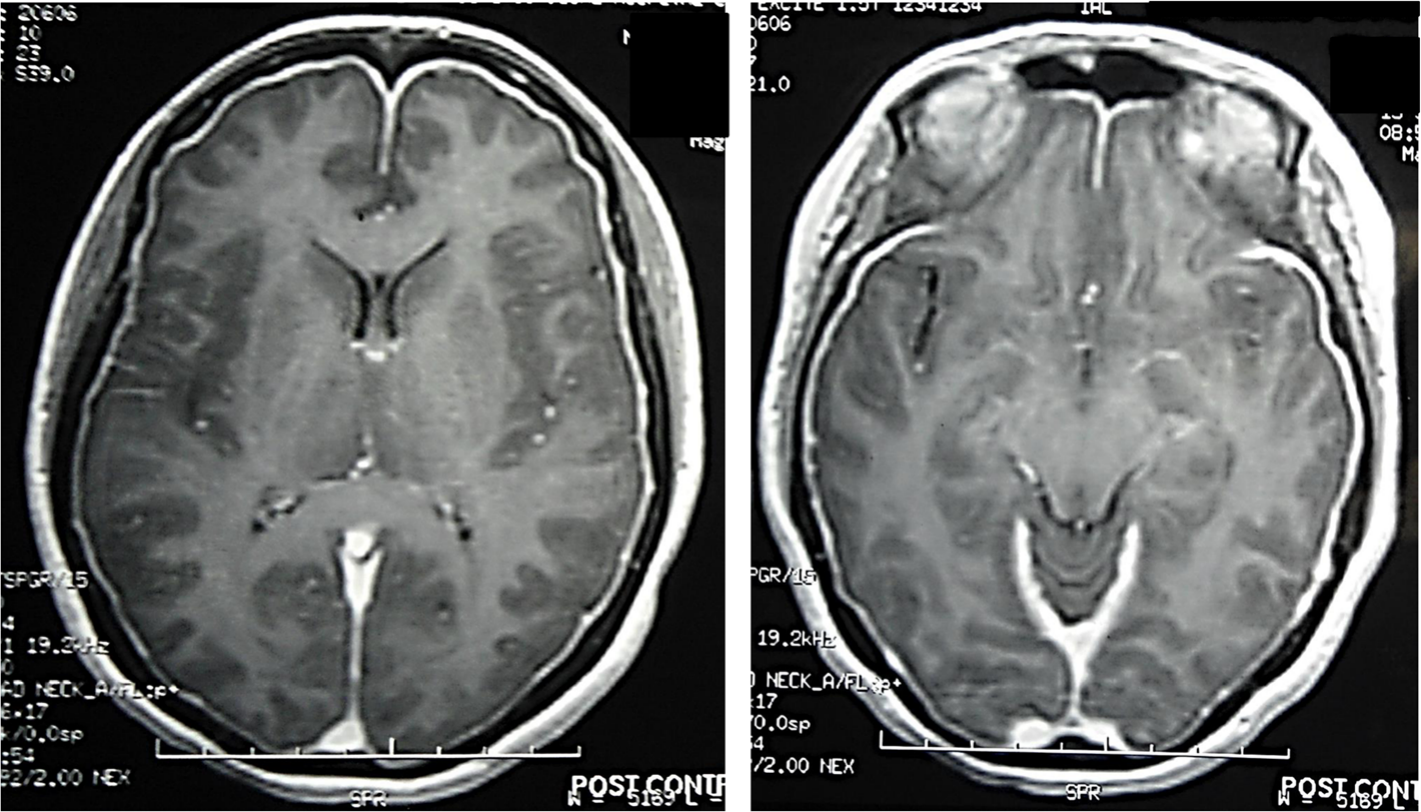
**Gadolinium-enhanced cranial magnetic resonance imaging showing generalized, uniform pachymeningeal enhancement.**

Haematological and biochemical blood investigations including full blood count, electrolytes, random blood glucose, liver and renal function tests, erythrocyte sedimentation rate and C-reactive protein were normal. Electrocardiogram was normal. Computerised tomography (CT) scan of the head did not reveal any abnormality. However, gadolinium-enhanced magnetic resonance imaging (MRI) showed generalized meningeal enhancement (Figure 1). The MR angiogram was normal.

Lumbar puncture done in the lateral decubitus position revealed a CSF opening pressure of less than 30 mm of H_2_O. The biochemical, cytological and microbiological analysis of CSF was normal and there was no xanthochromia. MR myelography failed to identify the site of CSF leak.

The patient was managed with bed-rest and hydration with infusions of normal saline. She was prescribed analgesics and encouraged to drink excess amounts of coffee ad libitum. Over the ensuing 3 months, her headaches became less intense and she could progressively tolerate longer durations in the upright posture. At three months’ review she was able to maintain her upright posture for up to 6 hours without headache. Since she showed small but definite improvement each day, the plan for epidural blood patching (EBP) was perpetually deferred. However, in retrospect, given the protracted time to recovery it would have been appropriate to have instituted EBP earlier.

## Conclusions

Our case report illustrates the rare but typical syndrome of spontaneous intracranial hypotension (SIH) characterized by positional orthostatic headache and pachymeningeal enhancement on neuroimaging that led to the discovery of a low CSF opening pressure (<60 mmH_2_0). Low pressure headache following dural puncture rarely presents diagnostic difficulty, but when it occurs spontaneously as in our patient misdiagnosis is the rule. Although the headache is typically orthostatic, other patterns have been reported ranging from chronic daily headaches, intermittent headaches, paradoxical headache that worsens on recumbency and headaches that mimic primary cough/exertional headache [4]. Interestingly, our patient presented with a thunderclap headache, which is a rare but recognised manifestation of SIH [5,6], further highlighting how easily it could be misdiagnosed. In addition to headache, patients may develop neck pain, nausea, vomiting, hyperacusis, tinnitus, unsteadiness, visual obscurations and abducens nerve palsies.

In SIH, the prevailing aetiology for low CSF pressure is considered to be CSF leakage located in the spine, most commonly in the throracic or cervicothorcic junction. In many instances of reported literature, SIH occurs in previously healthy individuals which indicate an acute event triggering a CSF leak [7]. A tear of the intricate meningeal coverings of nerve root sleeves emanating from spinal cord or a rupture of meningeal diverticula (reportedly in individuals with connective tissue disorders such as Marfan syndrome) or rupture of spinal epidural or perineural cysts may be the source of the cryptic CSF leak [8]. The disruption of meningeal continuity can be triggered by a trivial event such as a minor fall, a sudden twist or stretch, sexual intercourse, a sudden sneeze or vigorous exercise, which the patient often fails to recall as the inciting event [7]. Traction on pain-sensitive intracranial and meningeal structures because of the CSF hypovolaemia, particularly sensory nerves and bridging veins, is thought to cause headache and some of the associated symptoms. In the upright position this traction is exaggerated, hence the postural component of the headache. Secondary vasodilation of the cerebral vessels to compensate for the low CSF pressure may contribute to the vascular component of the headache by increasing brain blood volume. Because jugular venous compression increases headache severity, it seems likely that venodilation is also a contributing factor to the headache.

Diagnosis is based on the demonstration of low CSF pressure (<60 mmH_2_0) in a patient presenting with a postural orthostatic headache that is not better accounted by an alternative diagnosis [9]. The advent of MRI has greatly improved diagnosis although up to 20% may have a normal MRI [2]. The acronym SEEPS (for Subdural fluid collections, Enhancement of the pachymeninges, Engorgement of the venous structures, Pituitary enlargement, and Sagging of the brain) recalls the major features of SIH on brain MRI. CT myelography, radioisotope cisternography and MR myelography are utilized to identify the site of leak.

Epidural blood patching (EBP) is the mainstay of treatment and recommended for severe disabling SIH and in patients not responding to conservative management. It is hypothesized that EBP works initially through tamponade of the dural leak, and later by fibrin deposition that occurs within about three weeks [10]. Lumbar placement of the EBP can be effective even when the site of CSF leakage is above the site of the blood patch or is unknown. However, many patients may require more than one EBP treatment for a successful outcome [11]. Conservative management is recommended for mild to moderate SIH and includes avoidance of the upright posture, analgesics and strategies to increase CSF volume such as intravenous hydration, high caffeine intake and high salt intake. Oral or intravenous caffeine which is an adenosine receptor antagonist that increases CSF production as a secondary effect to reducing intracerebral blood flow, is of proven efficacy against post lumbar puncture headaches [12]. It is also used as therapy in SIH although its efficacy for this indication is unproven.

SIH is a rare and treatable cause of disabling headache and our case highlights a rare presentation of this syndrome. Awareness of its varied spectrum of clinical and radiographic manifestations is essential to avoid inappropriate investigations, misinterpretation of imaging results and unnecessary, ineffective treatment.

## Consent

Written informed consent was obtained from the patient for publication of this Case report and the accompanying images. A copy of the written consent is available for review by the Editor of this journal.

## Abbreviations

CSF: Cerebrospinal fluid
CT: Computerized tomography
EBP: Epidural blood patching
MR: Magnetic resonance
MRI: Magnetic resonance imaging
SIH: Spontaneous intracranial hypotension
